# Machine learning-based methods in diagnosing cardiac amyloidosis: a meta-analysis

**DOI:** 10.3389/fcvm.2026.1835652

**Published:** 2026-07-03

**Authors:** Yuchen Song, Qun Wang, Lianqun Jia, Yupeng Pei

**Affiliations:** 1 College of Integrated Chinese and Western Medicine, Liaoning University of Traditional Chinese Medicine, Shenyang, Liaoning, China; 2 Key Laboratory of Ministry of Education for TCM Viscera-State Theory and Applications, Ministry of Education of China, Liaoning University of Traditional Chinese Medicine, Shenyang, Liaoning, China; 3 Benxi Campus Management Committee, Liaoning University of Traditional Chinese Medicine, Shenyang, Liaoning, China

**Keywords:** cardiac amyloidosis, diagnosis, echocardiography, light chain amyloidosis, machine learning, meta-analysis, transthyretin cardiac amyloidosis

## Abstract

**Background:**

Cardiac amyloidosis (CA) is an infiltrative restrictive cardiomyopathy characterized by the deposition of *β*-fold amyloid, often presenting as left ventricular hypertrophy. Early nonspecific symptoms lead to frequent misdiagnosis as hypertrophic cardiomyopathy, delaying care for this progressive disease. While machine learning (ML) has been applied to the diagnosis of CA, systematic evidence of its accuracy remains lacking, hindering the development of intelligent detection tools.

**Objectives:**

To explore the diagnostic accuracy of ML, providing evidence-based data to advance smart detection tools for CA.

**Methods:**

We searched the Cochrane Library, PubMed, Embase, and Web of Science up to September 25, 2025, adhering to PRISMA 2020 guidelines. Study quality was evaluated using the QUADAS-2 instrument. Subgroup analyses were stratified by disease type [light chain CA (AL-CA), transthyretin CA (ATTR-CA)] and imaging modality (echocardiography) to explore sources of heterogeneity and assess diagnostic performance across different clinical scenarios.

**Results:**

The current meta-analysis incorporated 30 studies. In validation sets, ML for overall CA showed sensitivity 0.87 [95% confidence interval (CI) 0.83–0.91], specificity 0.88 (95% CI: 0.81–0.92), positive likelihood ratio (PLR) 7.0 (95% CI: 4.4–11.4), negative likelihood ratio (NLR) 0.14 (95% CI: 0.10–0.20), and SROC AUC 0.93 (95% CI: 0.91–0.95). For AL-CA, ML demonstrated sensitivity 0.85 (95% CI: 0.76–0.91), specificity 0.82 (95% CI: 0.75–0.87), PLR 4.8 (95% CI: 3.4–6.7), NLR 0.18 (95% CI: 0.11–0.30), and SROC AUC 0.88 (95% CI: 0.85–0.91). For ATTR-CA, ML revealed sensitivity 0.84 (95% CI: 0.77–0.89), specificity 0.85 (95% CI: 0.78–0.91), PLR 5.7 (95% CI: 3.6–9.2), NLR 0.19 (95% CI: 0.12–0.28), and SROC AUC 0.91 (95% CI: 0.88–0.93). Echocardiography-only ML models showed sensitivity 0.83 (95% CI: 0.81–0.85), specificity 0.86 (95% CI: 0.82–0.89), PLR 5.9 (95% CI: 4.4–7.9), NLR 0.20 (95% CI: 0.17–0.23), and SROC AUC 0.88 (95% CI: 0.85–0.91).

**Conclusions:**

ML demonstrates favorable diagnostic accuracy for CA. Nevertheless, the aggregated findings warrant cautious interpretation owing to inherent methodological limitations in the existing evidence. Future investigations incorporating diverse cases from broader geographic regions are needed to further validate the diagnostic performance of ML for CA and to advance the subsequent development of assessment tools based on artificial intelligence.

**Systematic Review Registration:**

PROSPERO CRD42024536601.

## Introduction

Cardiac amyloidosis (CA) is a critical and progressive infiltrative condition resulting from the deposition of amyloidogenic fibers in the heart (1). The disease is driven by the buildup of insoluble aggregates of misfolded proteins in tissues; the extracellular deposition of these amyloidogenic fibrils in the myocardium ultimately culminates in CA (2). According to a relevant study, the prevalence of CA in Germans increased from 15.5/100,000 person-years to 47.6/100,000 person-years, and the incidence rate rose from 4.8/100,000 person-years to 11.6/100,000 person-years over a 10-year observation period. Notably, the prevalence is still rising. Moreover, a marked elevation is noticed in the age and the proportion of males among the patients with amyloidosis (3). In CA patients, if left untreated, misfolded proteins deposit into the myocardial extracellular space, leading to thickened and hardened ventricular walls—a process that can induce heart failure, conduction dysfunction, or even death (4). Therefore, the early identification of cardiac degeneration is of profound clinical significance.

The diagnostic methods for CA have been classified as invasive and non-invasive in clinical practice. Invasive methods are applicable to all variants of CA, while non-invasive approaches are limited to transthyretin cardiac amyloidosis (ATTR-CA) (5). Among the invasive diagnostic methods, CA can be diagnosed when an endomyocardial myocardial biopsy exhibits deposition of amyloid after being stained with Congo red. In addition to suspicion raised by cardiac and extracardiac tests, CA should always be considered when cardiac patients suffer from typical systemic conditions, including plasma cell malignancy, nephrotic syndrome, peripheral neuropathy, or chronic systemic inflammatory diseases (1).

CA can be clinically diagnosed through imaging and endomyocardial myocardial biopsies. Diagnostic imaging has developed into a targeted tool for patients with suspected CA, like echocardiography, cardiac magnetic resonance (CMR), and bone scintigraphy (BS). Notably, CMR offers extensive cardiac data, including anatomical, functional, and tissue features, and its results demonstrate high sensitivity and specificity in diagnosing CA (6). In addition, cardiac biopsy, a highly invasive procedure, is extensively utilized in diagnosing CA. However, it is used only when amyloidosis is highly suspected. Unfortunately, CA is not usually symptomatic until later in life, and even at that stage, the symptoms are often quite nonspecific (7). Therefore, finding an efficient aid to help diagnose CA is of great significance.

With the swift progress in computing and telecommunications in recent years, wearable devices, such as electrocardiogram (ECG)-enabled smartwatches and single-lead wireless ECG patches, have demonstrated the potential of artificial intelligence (AI) for monitoring cardiovascular events (8). These devices are not only easy to wear but also show high sensitivity and specificity to diseases (9). Consequently, AI, represented by machine learning (ML), is found to be very helpful in diagnosing patients' conditions (10–12). Some investigators have sought to implement ML to diagnose CA (13–15). Nevertheless, there is no systematic evidence regarding its accuracy. This presents both a promising opportunity and an unprecedented challenge for intelligent diagnosis of CA. Hence, we perform this systematic review and meta-analysis to examine the diagnostic effectiveness of ML for CA and to provide effective evidence-based data for advancing intelligent diagnosis in this field.

## Methods

### Study registration

The current study was executed following the reporting guidelines for systematic reviews and meta-analyses (PRISMA 2020) and was prospectively registered with PROSPERO (CRD42024536601).

### Eligibility criteria

We established comprehensive eligibility criteria (Table 1) before the literature search.

**Table 1:** Eligibility criteria for original studies screened for this systematic review.

Items	Inclusion criteria	Exclusion criteria
P (Populations)	General population	None
E (Exposure)	Studies involving the complete development of a machine learning model (MLM) for CA diagnosis, with no restrictions on the types of predictor variables;	(1) Studies that only performed differential factor analysis without developing a complete MLM; (2) Studies that only performed image segmentation without constructing an MLM for identifying CA.
C (Control)	The control group for our study comprised non-CA subjects, including healthy individuals or patients with other cardiac conditions in the differential diagnosis of CA.	None
O (Outcomes)	The outcome measures were those used to evaluate model performance, including the receiver operating characteristic curve (ROC), c-statistic, c-index, sensitivity, specificity, accuracy, recall, precision, confusion matrix, diagnostic four-cell table, F1 score, and calibration curve.	Research lacking any outcome measures to assess the model accuracy.
S (Study design)	(1) Case-control studies, cohort studies, cross-sectional studies, and randomized controlled trials; (2) A limited number of studies employed only internal validation techniques (e.g., cross-validation or bootstrap validation). Despite the lack of an independent external validation set, these studies were included given the importance of evaluating overfitting in ML; (3) A small number of studies may be based on different ML studies published in the same open database. These studies were also included; (4) Research published in English.	(1) Meta-analyses, reviews, guidelines, and expert opinions; (2) Studies with a sample size of fewer than 20 total cases. This criterion ensures compliance with the Events Per Variable (EPV) rule of thumb of >10 for multivariate MLM development to mitigate overfitting. As a minimum of two predictor variables is expected in such models, a lower limit of 20 cases was set.

### Data sources and search strategy

We systematically searched PubMed, Cochrane, Embase, and Web of Science up to September 25, 2025. The search strategy employed both subject words and free words, without restricting publication region or year. Details are presented in Supplementary Appendix 1.

### Study selection and data extraction

The screened and selected studies were imported into the EndNote software. After eliminating duplicates, title and abstract screening was then executed. Subsequently, the full texts of the original studies were downloaded and read. Before carrying out data extraction, a spreadsheet was specified for basic information extraction, which included 23 categories of items such as study title, first author, time of publication, type of study, source of patients, type of task, control disease, diagnostic criteria and number of CA cases, and modeling method. The process of study screening was executed independently by two investigators (y.c.S and y.p.P) and then cross-checked, with a third investigator (l.q.J) assisting in adjudicating any disputes.

### Risk of bias (RoB) assessment

RoB of the included studies was assessed by means of a diagnostic trial evaluation tool, QUADAS-2, which evaluated the RoB and clinical applicability of original diagnostic trials (16). QUADAS-2 consisted of four domains: Patient Selection, Index Test, Reference Standard, and Flow and Timing. Each domain contained several specific questions, which were answered by “yes”, “no”, or “uncertain”. If all responses to the landmark questions within a domain were “yes”, the RoB could be assessed as low. However, if any answer to the informative questions was “no”, the RoB could be assessed as high. Moreover, investigators must then evaluate RoB based on the established guidelines. Uncertain RoB was graded as insufficient detail provided in the included studies hindered investigators from making a judgment.

Two investigators (y.c.S and y.p.P) independently evaluated RoB utilizing QUADAS-2, and their results were cross-checked. A third investigator (l.q.J) was invited to assist in judgment if there was a dispute.

### Synthesis methods

The meta-analysis of sensitivity and specificity was based on the diagnostic quadrangle table and conducted on the validation set. However, the majority of original studies failed to report the table. When absent, the table was reconstructed using sensitivity, specificity, positive predictive value, and accuracy, combined with the number of cases in this case (Equations 1–4). A bivariate mixed-effects model was leveraged to pool sensitivity, specificity, PLR, NLR, DOR, and SROC AUC. A nomogram was employed to assess the clinical applicability of the meta-analysis results. Meta-analysis was done in Stata 17.0.

TP=Sensitivity×NumberofCAcases (1)

TN=Specificity×Numberofnon-CAcases (2)

FP=Numberofnon-CAcases−TN (3)

FN=NumberofCAcases−TF (4)

## Results

### Study selection

**Figure 1:**
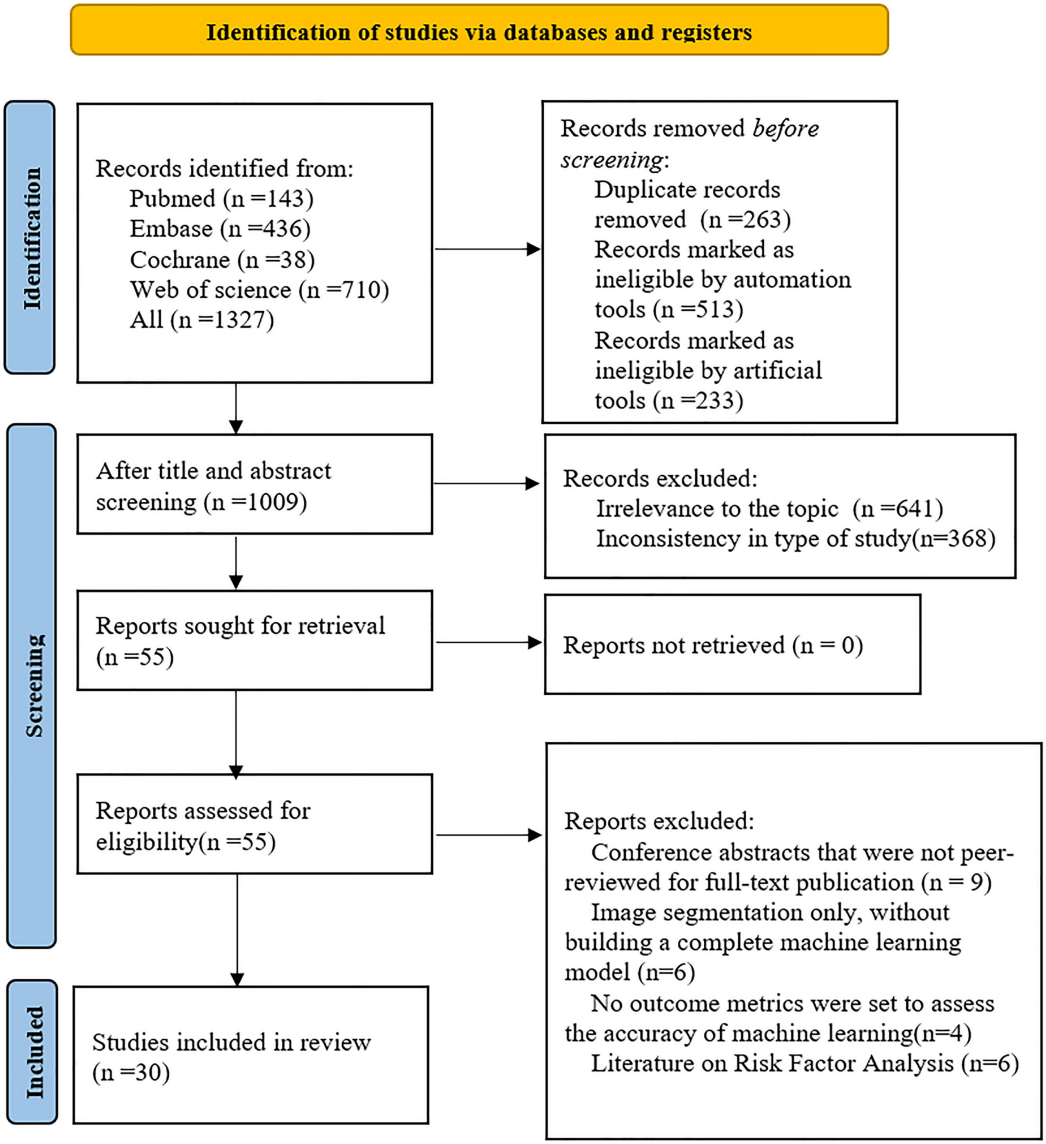
PRISMA flowchart of the literature screening process.

The literature retrieval yielded 1,327 articles, including 143 from PubMed, 436 from Embase, 38 from Cochrane, and 710 from Web of Science. Of these, 263 duplicates were deleted, and 1,009 articles were excluded owing to unrelated topics (*n* = 641) and inconsistent study types (*n* = 368). The full texts of the remaining 55 articles were retrieved and reviewed for eligibility. A further 25 articles were eliminated for the following reasons: conference abstracts lacking full-text publication or peer review (*n* = 9), studies solely addressing segmentation of images without a complete ML model (*n* = 6), studies not reporting outcome metrics for evaluating the accuracy of ML (*n* = 4), and risk factor analyses (*n* = 6). Finally, 30 original studies were included (17–46). The detailed search strategy is available in Figure 1.

### Study characteristics

This meta-analysis incorporated 30 original studies (17–46) conducted from 2018 to 2025. Fifteen of these studies were case-control studies. Seven originated from China (17, 19, 20, 23, 29, 43, 46), eight from the United States (18, 24, 25, 31, 33, 42, 44, 45), three from Italy (22, 32, 39), three from France (26, 27, 30), two from Australia (21, 36), one from Brazil (38), one from Greece (35), two from the United Kingdom (37, 40), one from Germany (28), one from Japan (41), and one from Spain (34). Eleven of these studies were single-center (17, 19, 22, 23, 26–28, 32, 36, 41, 42), seven were multicenter (20, 24, 29–31, 33, 34), and twelve were database studies (18, 21, 25, 35, 37–40, 43–46). The eligible studies encompassed 19,689 subjects with CA. Twelve studies utilized external validation sets (18, 20, 24, 25, 29–31, 33, 34, 40, 43, 45), eight employed random sampling for validation (17, 20, 23, 26, 35, 37, 42, 46), and three implemented cross-validation (22, 27, 36). Regarding modeling, six studies selected echocardiography (17, 18, 25, 31, 40, 42), four used electrocardiography (21, 22, 24, 25), eight employed CMR (19, 20, 26–29, 35, 36), and one used BS for presentation (30). In the field of ML, thirteen studies employed deep learning (DL) techniques (18, 24–27, 29, 30, 36, 39–41, 44, 45). Only two of the studies included in this review compared their results with those of clinical practitioners (21, 37) (Table 2 and Supplementary Appendix 2).

**Table 2:** Basic characteristics of original studies included in this systematic review.

No.	First Author	Year of publication	Author country	Patient source	Control disease	Number of cardiac amyloidosis cases	Total number of cases	Generation methods of validation set	Model type	Modeling images	Whether to be compared with clinicians
1	Zhang X (17)	2023	China	Single center	Hypertrophic cardiomyopathy; Uremic cardiomyopathy; Hypertensive heart disease	P:50	P:289	Random sampling	LR/RF/SVM/GBDT	echocardiography	No
2	Zhang J (18)	2018	USA	Registry database	Hypertrophic cardiomyopathy and pulmonary hypertension	I:804	I:14,035	External validation	DL	echocardiography	No
3	Yue X (19)	2022	China	Single center	Patients with hypertrophic cardiomyopathy and healthy controls.	P:38	P:71		LR	Cardiovascular magnetic resonance	No
4	Zhou XY (20)	2022	China	Multi-center	unclear	P:110	P:200	Random sampling, External validation	XGBoost	Cardiovascular magnetic resonance	No
5	Schrutka L (21)	2021	Austria	Registry database	Heart failure, hypertrophic cardiomyopathy	I:271	I:382		KNN	Electrocardiogram	Yes
6	Lo Iacono F (22)	2023	Italy	Single center	Aortic stenosis	P:15	P:30	Cross-validation	KNN/SVM/DT/LR/GBM	CCT/CT	No
7	Huang S (23)	2022	China	Single center	Hypertrophic cardiomyopathy (HCM)	P:100	P:317	Random sampling	RF		No
8	Haimovich JS (24)	2023	USA	Multi-center	Hypertrophic cardiomyopathy	P:304	P:50,709	External validation	DL	Electrocardiogram	No
9	Goto S (25)	2021	USA	Registry database	Hypertrophic cardiomyopathy; hypertension	P:3,026	P:12,878	External validation	DL	Electrocardiogram and echocardiography	No
10	Germain P (26)	2022	France	Single center	Left ventricular hypertrophy	P:119	P:241	Random sampling	DL	Cardiac magnetic resonance	No
11	Germain P (27)	2023	France	Single center	AL/CMR	P:70	P:120	Cross-validation	DL	MRI	No
12	Eckstein J (28)	2022	Germany	Single center	Hypertrophic cardiomyopathy	P:43	P:63		DT/KNN/SVM/RBF	MRI	No
13	Diao K (29)	2023	China	Multi-center	HCM or HHD	P:69	P:355	External validation	DL	MRI	No
14	Delbarre MA (30)	2023	France	Multi-center		I:383	I:4,681	External validation	DL	Whole body bone scintillation imaging	No
15	Cuddy SA (31)	2022	USA	Multi-center	ATTR-CA	P:324	P:598	External validation	LR	echocardiography	No
16	Barbieri A (32)	2023	Italy	Single center	Healthy people, HCM patients, isolated hypertension	P:10	P:187		LR		No
17	Arvanitis M (33)	2017	USA	Multi-center	Heart failure	P:27	P:77	External validation	LR		No
18	Arana-Achaga X (34)	2023	Spain	Multi-center	Hypertensive cardiomyopathy, AS	P:108	P:227	External validation	LR		No
19	Antonopoulos AS (35)	2021	Greece	Registry database	Left ventricular hypertrophy	P:28	P:152	Random sampling	LR	Cardiac magnetic resonance	No
20	Martini N (36)	2020	Italy	Single center	Monoclonal *γ* disease or unexplained increase in left ventricular wall thickness on echocardiography	P:107	P:206	Random sampling	DL	Cardiovascular magnetic resonance	No
21	Tsang C (37)	2023	UK	Registry database	Heart failure		P:15,008	Random sampling			Yes
22	Zuppo Laper I (38)	2024	Brazil	Registry database		P:1,483	P:1,506,361		LR/SVM/XGBoost/RF		No
23	Bargagna F (39)	2025	Italy	Registry database	CA; AL-CA; ATTR-CA	AL-CA:13 ATTR-CA:15	I:47	K-fold validation	DL	PET	No
24	Slivnick JA (40)	2025	UK	Registry database	CA	P:1,359	P:2,612	External validation	DL	Echocardiography	No
25	Tohyama T (41)	2025	Japan	Single center	CA	P:76	P:90		DL	HE image	No
26	Chang RS (42)	2024	USA	Single center	CA	P:231	P:636	Random sampling	RF	Echocardiography	No
27	Hong Z (43)	2025	China	Registry database	CA; AL-CA; ATTR-CA	AL(*n* = 12) ATTR(*n* = 15)	P:50	External validation	LR	PET/CT	No
28	Vrudhula A (44)	2024	USA	Registry database	CA	P: 990 I: 10,042	P: 341,989 I: 1,344,372	Random sampling	DL	ECG	No
29	Hourmozdi J (45)	2025	USA	Registry database	ATTR-CA	P:176	P:3,368	External validation	RF/LR/DL		No
30	Pan Y (46)	2024	China	Registry database	CA	P:261	P:6,563	Random sampling	LR/RF/XGBoost		No

(1) In the model type, RF, random forest; SVM, support vector machine; GBDT, gradient boosting decision tree; KNN, K-nearest neighbors; DL, deep learning. (2) In the modeling images, CMR, cardiac magnetic resonance. (3) In the total number of cases, P, the number of patients, I, the number of images.

### Rob assessment

**Figure 2:**
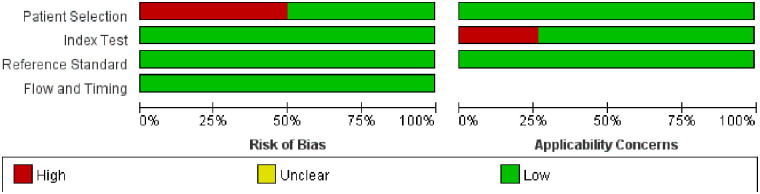
Summary of QUANDAS-2 risk of bias assessments for included original studies.

**Figure 3:**
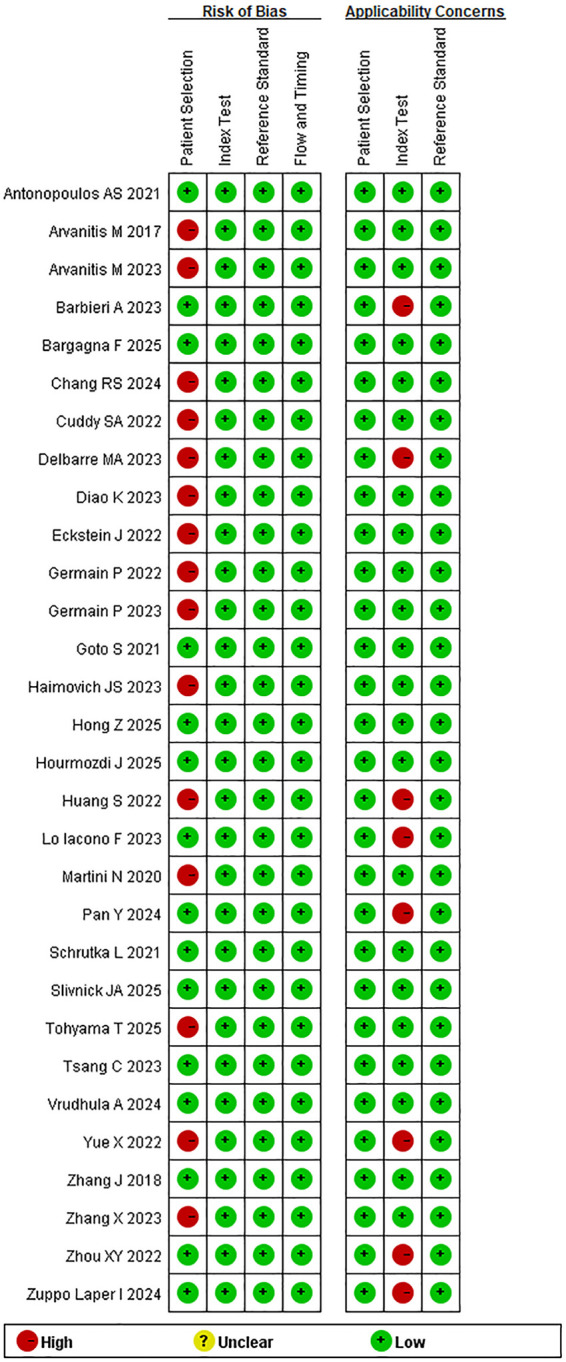
Detailed QUANDAS-2 risk of bias assessments for included original studies.

Fifteen of the 30 eligible studies were case-control studies from non-public databases, which introduced a high RoB in patient selection. Since ML assessment followed predefined algorithmic rules, it did not introduce a high RoB in the interpretation of the index test. In terms of the gold standard, it was feasible to accurately differentiate the state of disease. However, details regarding whether blinding was employed in disease evaluation were not thoroughly described. Therefore, the RoB in blinding could not be comprehensively assessed. In the flow of cases, all studies were rated as low risk, given that patients either received only one identical gold standard or there was an appropriate time interval between the trial under evaluation and the gold standard. Six studies only presented results for the training set, and 2 × 2 diagnostic tables could not be calculated in 2 studies due to insufficient case numbers. Consequently, the alignment between the implementation and interpretation of the index test and the review questions carried a high RoB. The comprehensive RoB assessment is depicted in Figures 2, 3.

### Meta-analysis

#### CA

**Figure 4:**
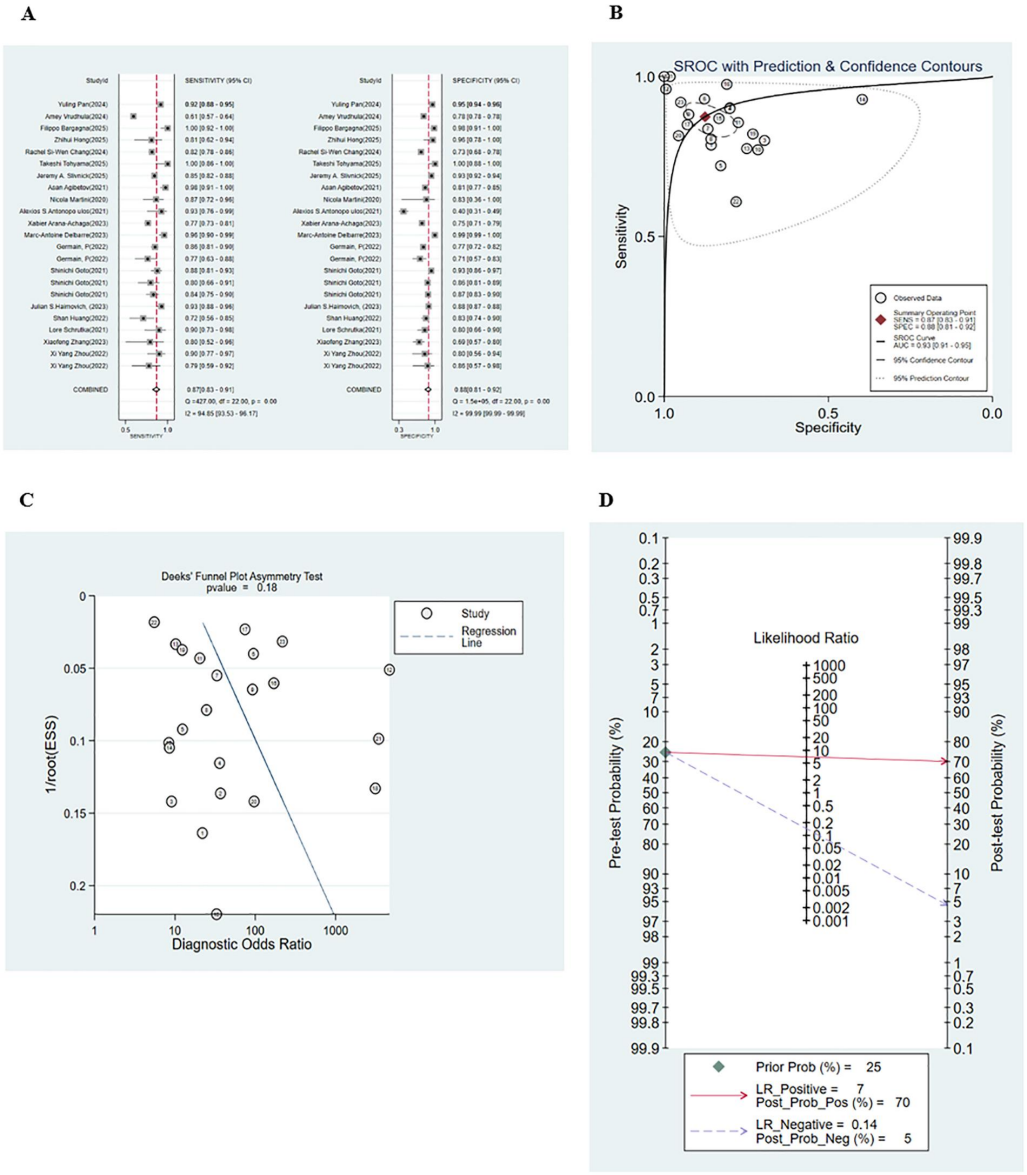
Meta-analysis results of machine learning for detecting cardiac amyloidosis. **(A)** Forest plot of meta-analysis for sensitivity and specificity; **(B)** summary receiver operating characteristic curve; **(C)** funnel plot for meta-analysis; **(D)** meta-analysis nomograms.

The meta-analysis was executed utilizing a bivariate mixed-effects model. The results demonstrated that in the validation set, respective sensitivity, specificity, PLR, NLR, DOR, and SROC AUC of ML for diagnosing CA were 0.87 (95% CI: 0.83–0.91), 0.88 (95% CI: 0.81–0.92), 7.0 (95% CI: 4.4–11.4), 0.14 (95% CI: 0.10–0.20), 49 (95% CI: 23–106), and 0.93 (95% CI: 0.91–0.95). Following the meta-analytic synthesis, the threshold effect was examined using Spearman's correlation coefficient, which yielded a value of 0.59. The pooled SROC curve displayed no shoulder-shaped pattern, indicating an absence of a notable threshold effect. Deeks' funnel plot indicated an absence of publication bias (*P* = 0.18). Assuming a prior high-risk probability of 25% for CA, the posterior probability of a patient having CA was 70% when the ML algorithm detected a positive result for CA. Conversely, a negative ML result for CA corresponded to a posterior probability of 5% for the presence of CA (Figure 4).

### External validation

**Figure 5:**
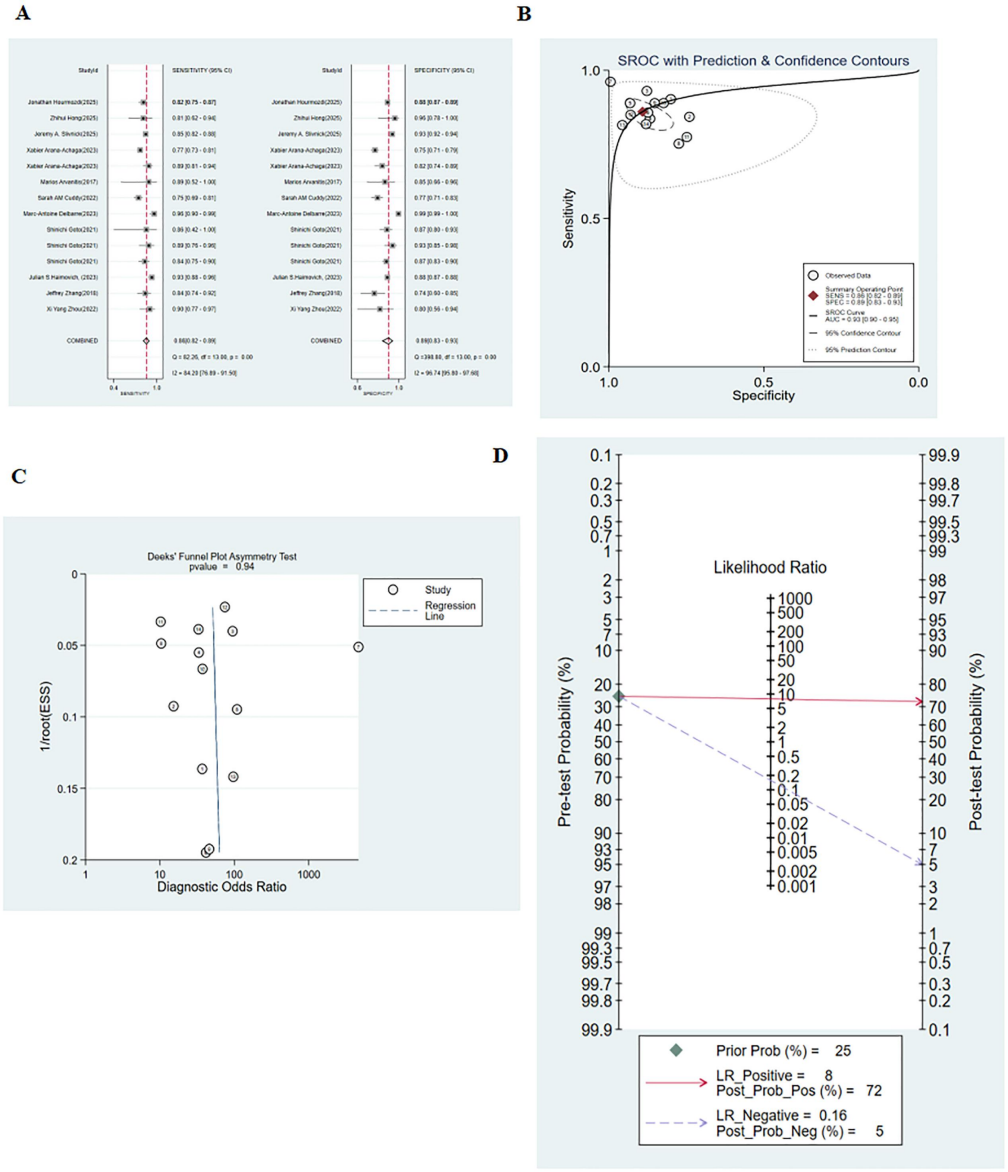
Meta-analysis results of machine learning for detecting light chain cardiac amyloidosis. **(A)** Forest plot of meta-analysis for sensitivity and specificity; **(B)** summary receiver operating characteristic curve; **(C)** funnel plot for meta-analysis; **(D)** meta-analysis nomograms).

In the external validation dataset, the meta-analysis was executed employing a bivariate mixed-effects model. The results demonstrated that in the validation set, the sensitivity, specificity, PLR, NLR, DOR, and SROC AUC of ML for diagnosing CA were 0.86 (95% CI: 0.82–0.89), 0.89 (95% CI: 0.83–0.93), 7.9 (95% CI: 4.7–13.2), 0.16 (95% CI: 0.12–0.21), 50 (95% CI: 23–110), and 0.93 (95% CI: 0.90–0.95), respectively. Deeks' funnel plot indicated an absence of publication bias (*P* = 0.94). Assuming a prior high-risk probability of 25% for CA, the posterior probability of a patient having CA was 72% when the ML algorithm detected a positive result for CA. Conversely, a negative ML result for CA corresponded to a posterior probability of 5% for the presence of CA (Figure 5).

### Registered database

**Figure 6:**
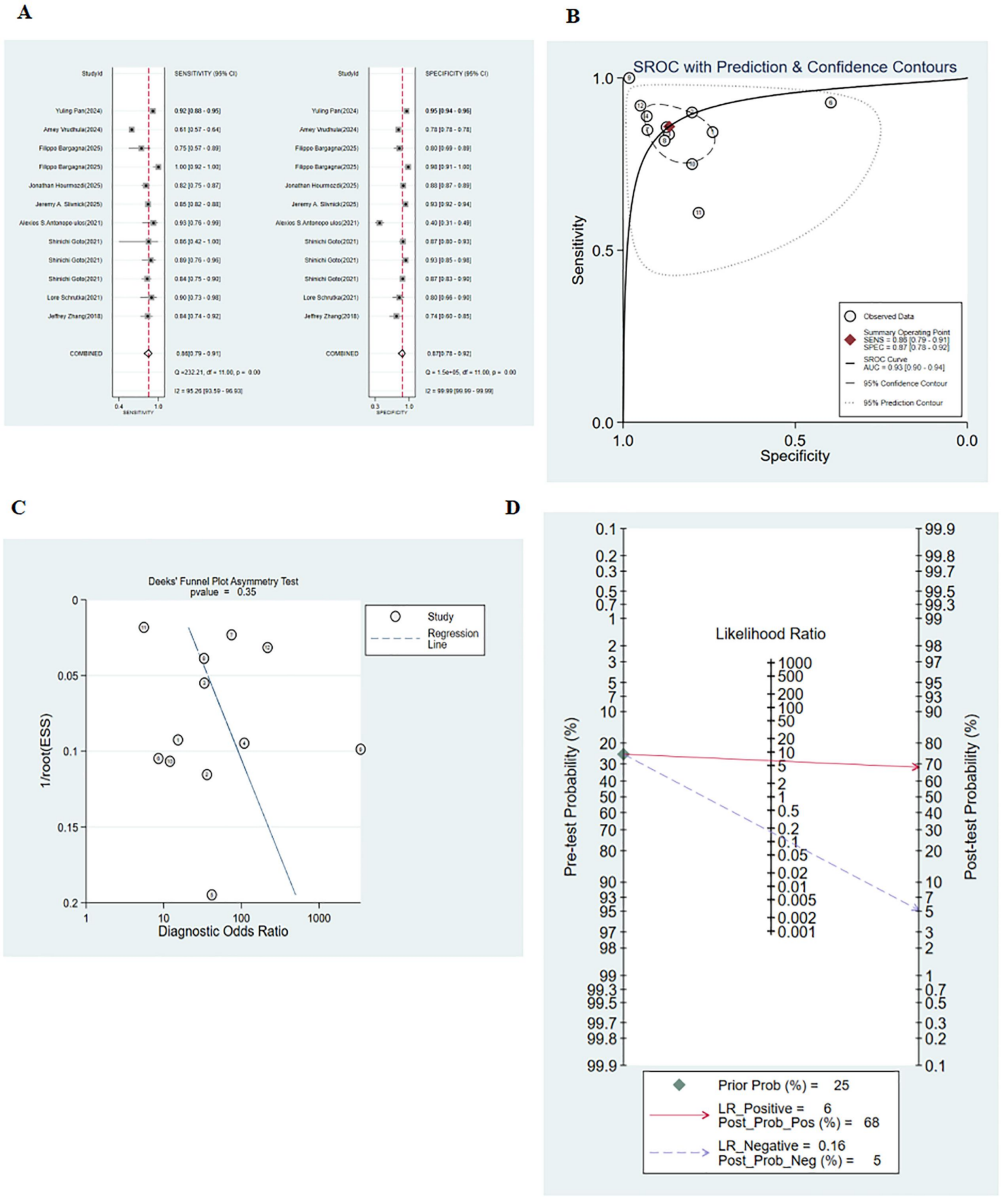
External validation of machine-learning for diagnosing cardiac electrical degeneration in validation set.

In the registered database, the meta-analysis was conducted via a bivariate mixed-effects model. The results demonstrated that in the validation set, the sensitivity, specificity, PLR, NLR, DOR, and SROC AUC of ML for diagnosing CA were 0.86 (95% CI: 0.79–0.91), 0.87 (95% CI: 0.78–0.92), 6.4 (95% CI: 3.8–11.0), 0.16 (95% CI: 0.10–0.25), 40 (95% CI: 16–96), and 0.93 (95% CI: 0.90–0.94), respectively. Deeks' funnel plot indicated an absence of publication bias (*P* = 0.35). Assuming a prior high-risk probability of 25% for CA, the posterior probability of a patient having CA was 68% when the ML algorithm detected a positive result for CA. Conversely, a negative ML result for CA corresponded to a posterior probability of 5% for the presence of CA (Figure 6).

### Individual-participant data

**Figure 7:**
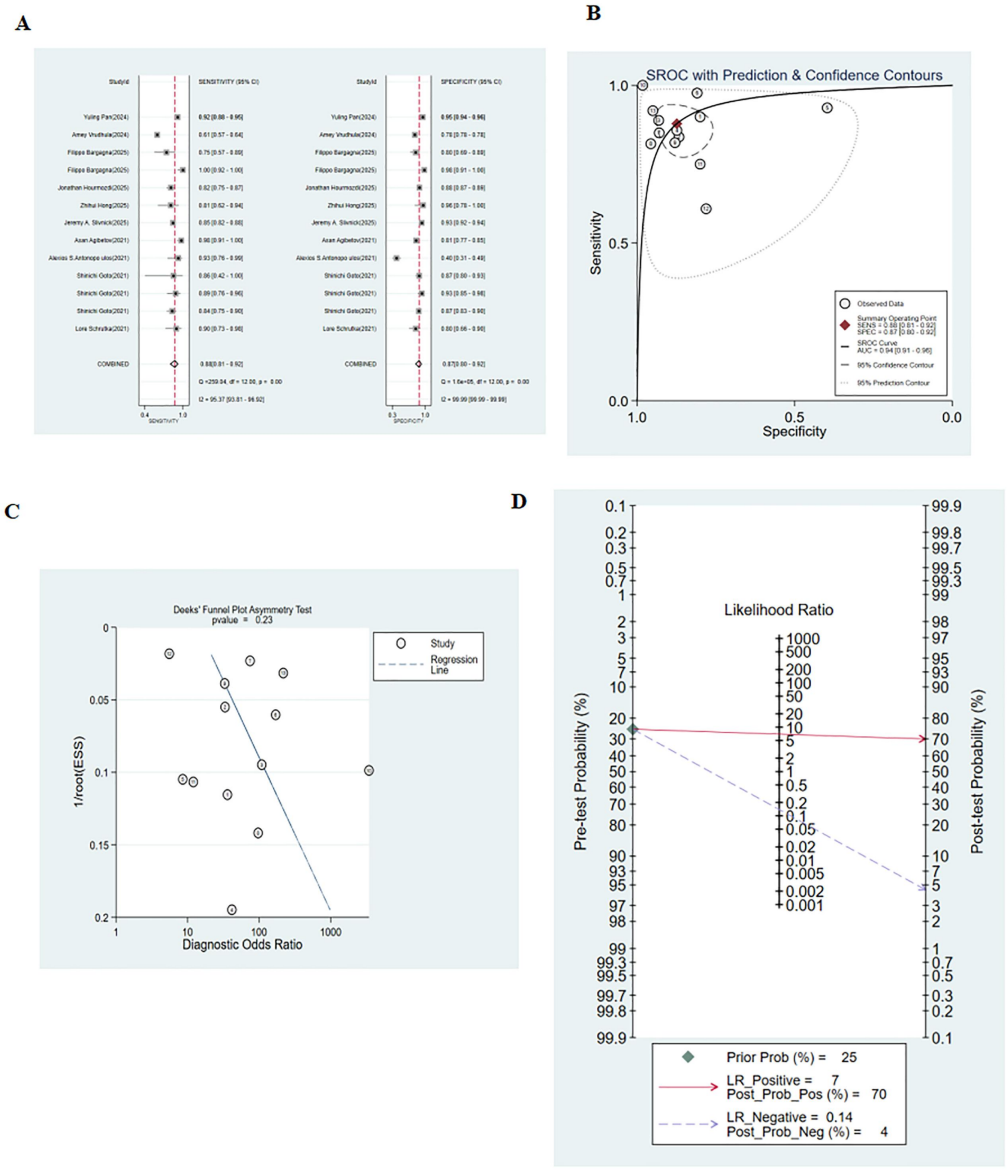
Meta-analysis results of machine-learning for detecting cardiac amyloidosis in registered dataset.

For individual-participant data, the meta-analysis was performed with a bivariate mixed-effects model. The results demonstrated that in the validation set, the sensitivity, specificity, PLR, NLR, DOR, and SROC AUC of ML for diagnosing CA were 0.88 (95% CI: 0.81–0.92), 0.87 (95% CI: 0.80–0.92), 7.0 (95% CI: 4.2–11.5), 0.14 (95% CI: 0.09–0.23), 50 (95% CI: 22–116), and 0.94 (95% CI: 0.91–0.96), respectively. Deeks' funnel plot indicated an absence of publication bias (*P* = 0.23). Assuming a prior high-risk probability of 25% for CA, the posterior probability of a patient having CA was 70% when the ML algorithm detected a positive result for CA. Conversely, a negative ML result for CA corresponded to a posterior probability of 4% for the presence of CA (Figure 7).

### Light chain CA (AL-CA)

**Figure 8:**
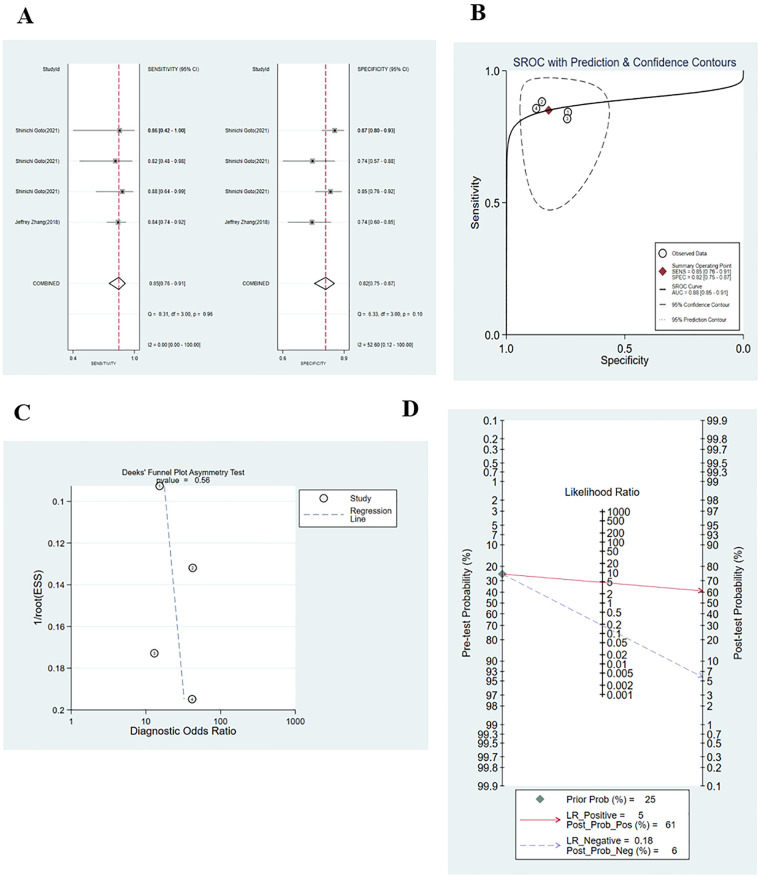
Meta-analysis results of machine-learning for detecting cardiac amyloidosis in individual-participant dataset.

The meta-analysis implied that the sensitivity, specificity, PLR, NLR, DOR, and SROC AUC of ML for diagnosing AL-CA were 0.85 (95% CI: 0.76–0.91), 0.82 (95% CI: 0.75- 0.87), 4.8 (95% CI: 3.4–6.7), 0.18 (95% CI: 0.11–0.30), 26 (95% CI: 13–54), and 0.88 (95% CI: 0.85–0.91), respectively. Deeks' funnel plot demonstrated an absence of publication bias (*P* = 0.56). Assuming a prior high-risk probability of 25% for AL-CA, the posterior probability of a patient having CA was 61% when the ML algorithm detected a positive result for AL-CA. Conversely, a negative ML result for AL-CA corresponded to a posterior probability of 6% for the presence of AL-CA (Figure 8).

### ATTR-CA

**Figure 9:**
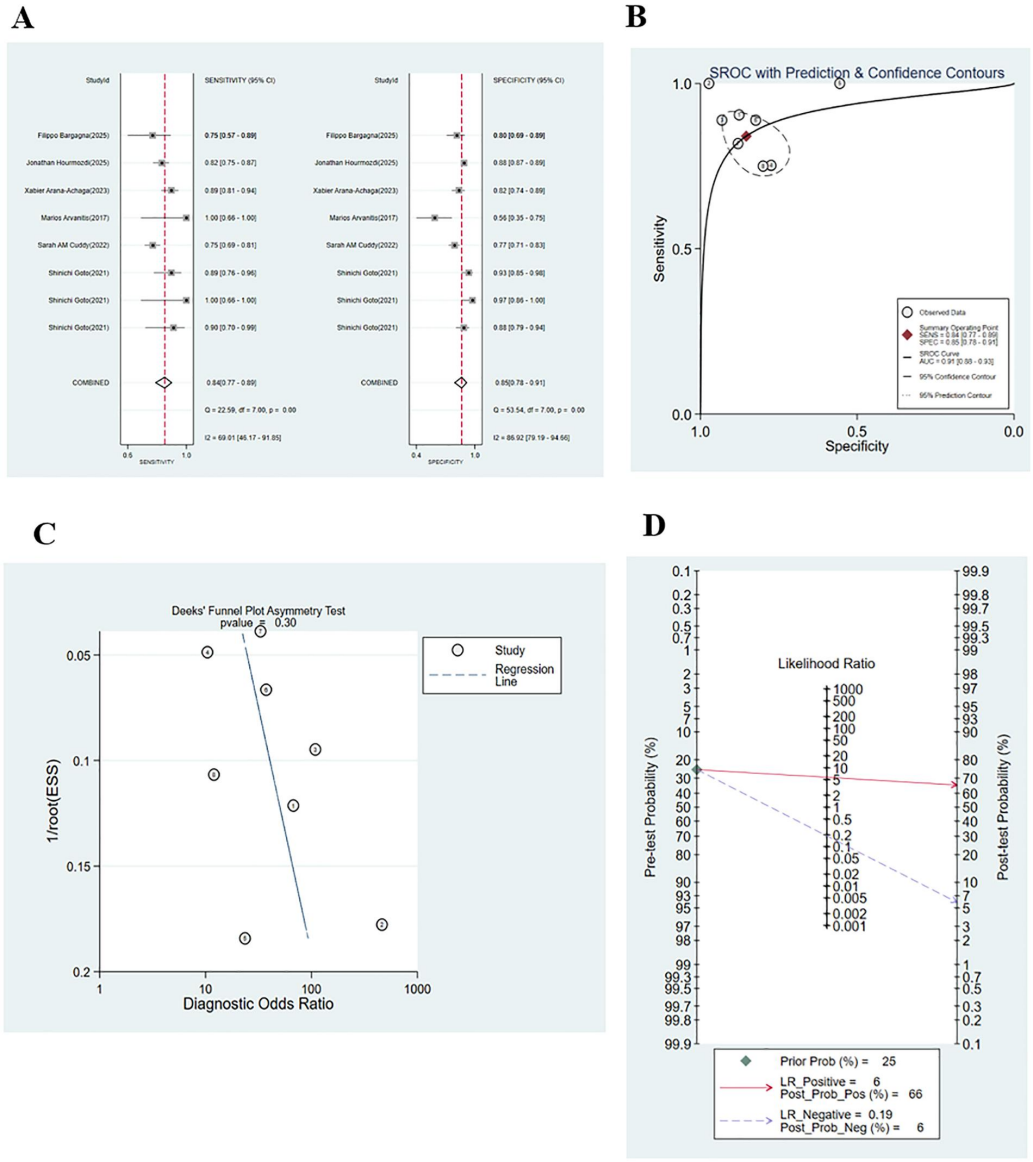
Meta-analysis results of machine learning for detecting transthyretin cardiac amyloidosis. (A) Forest plot of meta-analysis for sensitivity and specificity; **(B)** summary receiver operating characteristic curve; **(C)** funnel plot for meta-analysis; **(D)** meta-analysis nomograms).

The results revealed that the sensitivity, specificity, PLR, NLR, DOR, and SROC AUC of ML for diagnosing ATTR-CA were 0.84 (95% CI: 0.77–0.89), 0.85 (95% CI: 0.78- 0.91), 5.7 (95% CI: 3.6–9.2), 0.19 (95% CI: 0.12–0.28), 31 (95% CI: 14–69), and 0.91 (95% CI: 0.88–0.93), respectively. Deeks' funnel plot revealed an absence of publication bias (*P* = 0.07). Assuming a prior high-risk probability of 25% for ATTR-CA, the posterior probability of a patient having ATTR-CA was 66% when the ML algorithm detected a positive result for ATTR-CA. Conversely, a negative ML result for ATTR-CA corresponded to a posterior probability of 6% for the presence of ATTR-CA (Figures 6–9).

### Echocardiogram

**Figure 10:**
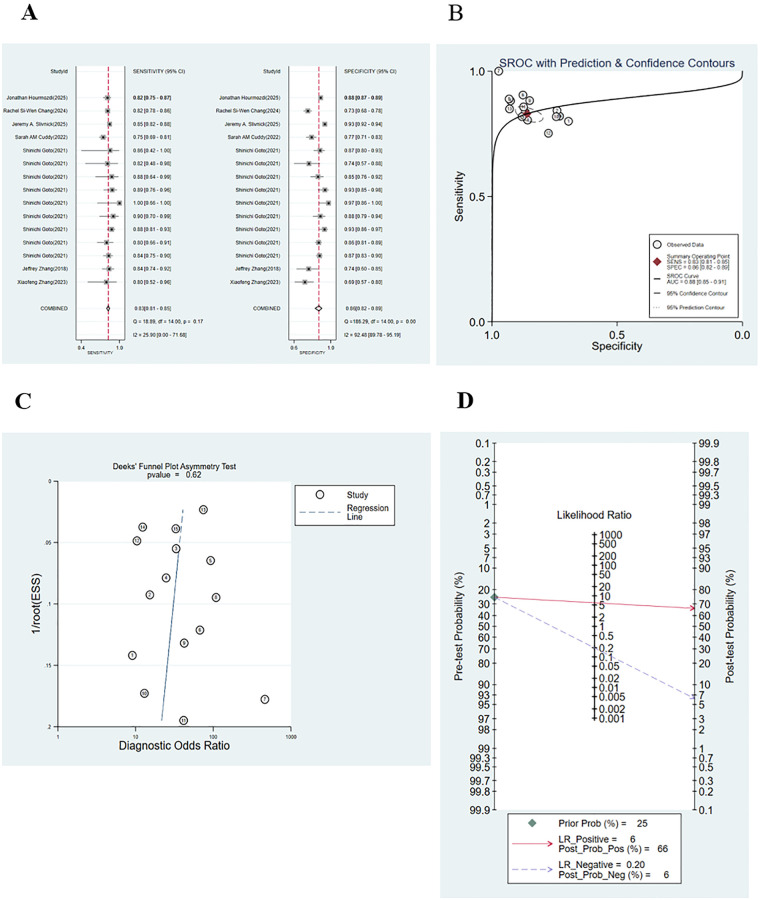
Meta-analysis results of machine learning based on echocardiography for detecting cardiac amyloidosis. **(A)** Forest plot of meta-analysis for sensitivity and specificity; **(B)** summary receiver operating characteristic curve; **(C)** funnel plot for meta-analysis; **(D)** meta-analysis nomograms).

Given the widespread use of echocardiography for detecting CA, the diagnostic effectiveness of echocardiography-based ML for CA was checked. The results showed that the sensitivity, specificity, PLR, NLR, DOR, and SROC AUC of ML were 0.83 (95% CI: 0.81–0.85), 0.86 (95% CI: 0.82–0.89), 5.9 (95% CI: 4.4–7.9), 0.20 (95% CI: 0.17–0.23), 30 (95% CI: 20–46), and 0.88 (95% CI: 0.85–0.91), respectively. Deeks' funnel plot suggested an absence of publication bias (*P* = 0.62). Assuming a prior high-risk probability of 25% for CA, the posterior probability of a patient having CA was 66% when the echocardiography-based ML algorithm detected a positive result for CA. Conversely, a negative echocardiography-based ML result for CA corresponded to a posterior probability of 6% for the presence of CA (Figure 10).

## Discussion

### Summary of main findings

This study found that ML is effective in diagnosing CA. In the validation set, the sensitivity and specificity of ML were 0.87 (95% CI: 0.83–0.91) and 0.88 (95% CI: 0.81–0.92) for diagnosing CA, 0.85 (95% CI: 0.76–0.91) and 0.82 (95% CI: 0.75- 0.87) for diagnosing AL-CA, 0.84 (95% CI: 0.77–0.89) and 0.85 (95% CI: 0.78- 0.91) for diagnosing ATTR-CA, and 0.83 (95% CI: 0.81–0.85) and 0.86 (95% CI: 0.82–0.89) for detecting CA based on echocardiography, respectively.

### Comparison with previous reviews

Studies have shown that echocardiography, electrocardiography, and CT scans are commonly used in clinical trials to diagnose CA (47–49). Semi-quantitative amyloid positron emission tomography (PET) parameters can help diagnose CA and identify its type (50). Kim et al. have utilized amyloid PET to diagnose CA. Their findings suggest that amyloid PET is a valuable method for diagnosing CA, with a sensitivity of 0.95 and a specificity of 0.98. Wu et al. have compared CMR, single photon emission computed tomography (SPECT), and PET modalities. They found sensitivities of 0.84, 0.98, and 0.78, and specificities of 0.87, 0.92, and 0.95 for the three modalities, respectively. These results demonstrate that SPECT scanning exhibits superior diagnostic performance in identifying CA (51). However, their investigation was based on a limited number of cases, and thus, the conclusions regarding the accuracy of SPECT for diagnosing CA remain tentative. Furthermore, SPECT entails considerable expense, posing a substantial challenge for economically underdeveloped regions with limited medical resources. Second, interpreting SPECT results demands substantial professional expertise, and interpretations may vary across clinical experts. Therefore, future research could explore using ML for CA diagnosis.

Furthermore, Aimo et al. have identified the prevalence of CA under various conditions. For instance, in studies related to non-cardiac causes of BS (*n* = 5), the prevalence of CA is 1% (0%, 1%). In cases of severe aortic stenosis (AS) (*n* = 7), the prevalence increases to 8% (5%, 13%). The researchers conclude that the mean age for CA patients varies between 74 and 90 years across different settings and that the percentage of male patients ranges from 50% to 100%. While ATTR-CA accounts for many cases in diverse environments, AL-CA is also not uncommon. These findings collectively demonstrate that the prevalence of CA is markedly high (52). According to Zhang et al., the N-terminal pro-B-type natriuretic peptide shows a sensitivity of 0.93 and a specificity of 0.84 for diagnosing cardiac involvement in CA patients, with an SROC AUC of 0.95. This offers a theoretical framework for clinical practice (53). Additionally, Jaiswal et al. suspect that AS and CA often coexist. By examining interventricular septal thickness (standardized mean difference: 0.74, 95% CI: 0.36–1.12, *P* = 0.0001), relative wall thickness (SMD: 0.74, 95% CI: 0.17–1.30, *P* < 0.0001), posterior wall thickness (SMD: 0.74, 95% CI: 0.51–0.97, *P* = 0.0011), and left ventricular mass index (SMD: 1.62, 95% CI: 0.63–2.62, *P* = 0.0014), they find that these indicators are notably elevated in AS-CA patients compared to those with AS only, providing a basis for diagnosing CA (54).

A shared pathological characteristic among all types of CA is the extracellular deposition of amyloid in the heart. In systemic AL-CA, this deposition is predominantly diffuse and occurs pericellularly, as well as within the endocardium and arterial structures, including small arteries, whereas ATTR-CA is primarily marked by nodular deposition patterns (47). AL-CA is attributed to a small B-cell clone, which produces toxic light chains that lead to the formation of amyloid deposits in tissues. These deposits primarily affect the heart and kidneys (55). Cardiomyopathy is predominantly attributed to systemic amyloidosis, which is caused by transthyretin (TTR) proteins. This condition arises from the deposition of TTR proteins. When TTR proteins misfold, they form amyloidogenic fibers that are deposited in the heart. This leads to heart failure, heart block, or arrhythmias, including atrial fibrillation (56). Our analysis revealed that ML achieved a sensitivity and specificity of 0.85 (95% CI: 0.76–0.91) and 0.82 (95% CI: 0.75–0.87) for diagnosing AL-CA, and of 0.84 (95% CI: 0.77–0.89) and 0.85 (95% CI: 0.78–0.91) for diagnosing ATTR-CA. Although prior work has demonstrated the accuracy of molecular-level approaches for diagnosing CA, these detection methods are invasive and necessitate a biopsy. They also entail considerable cost. Therefore, future research could use ML to diagnose CA, which would have positive clinical significance. DL methods demonstrate excellent performance across a range of classification and segmentation tasks. Bento et al. have applied DL to structural brain magnetic resonance imaging, concluding that these methods can explicitly address batch effects, resulting in consistent outcomes across various datasets (57). In addition, Spiller et al. have used supervised ML to monitor patient-specific drug responses by observing thermograms to explore morphological and textural features within death-like patient-derived organoids during drug therapy (58). Supervised ML performs well in both diagnosing the disease as well as predicting its prognosis (59). With the continuous development of ML, the accuracy of clinical imaging has been gradually improved. To construct a traditional radiomics-based ML model in a single study, typically, image segmentation should be first conducted, followed by image extraction, and finally model construction (60). Recently, image-based DL, particularly state-of-the-art unsupervised and semi-supervised approaches, has demonstrated advances in classification, segmentation, detection, and image alignment tasks for medical images (61). Notably, incorporating textures into training after completing image segmentation appears to mitigate information loss while enhancing predictive performance (62). Although few DL-based studies were included in this meta-analysis, a distinction remains between ML and DL. Therefore, future studies should focus on developing DL techniques for the intelligent detection of CA based on cardiac imaging to advance CA detection progress.

Our meta-analysis revealed that echocardiography was the primary imaging modality utilized for diagnosing CA, alongside CT and BS (63). Furthermore, echocardiography is regarded as the primary approach for evaluating suspected myocardial amyloid deposition (64). This meta-analysis indicated that echocardiography-based ML had a sensitivity and specificity of 0.83 (95% CI: 0.81–0.85) and 0.86 (95% CI: 0.82–0.89) for diagnosing CA. Therefore, this approach could be feasible for diagnosing CA in future clinical practice.

The variables used to construct the models in the included studies were primarily derived from imaging data (65), i.e., echocardiography, ECG, and CT. The validation sets were generated using internal and external validation, random sampling, and cross-validation (66). Nine studies employed external validation sets, seven studies used random sampling validation, and three studies utilized cross-validation. More external validation is needed in ML to prove its generalizability. Therefore, the present meta-analysis requires more external validation sets to illustrate its feasibility and performance (67, 68).

In recent years, numerous researchers have further explored the performance of ML and DL in modeling during the research process. AI, especially DL, has made significant progress in the field of medical image analysis (69), which has received extensive attention from researchers because DL can intelligently process and read images based on image modeling (70). In contrast, conventional ML relies on manual coding, resulting in slightly weaker performance in image processing. For conventional image processing, we recommend using DL. However, for constructing scoring tools, we prefer conventional ML because the process relies on interpretable clinical features (71). Although DL seems to be more advantageous for processing images, there are serious challenges in its interpretability. Thus, we prefer to use conventional ML based on clinical features.

Although 15 case–control studies derived from non-public databases were rated as carrying a high RoB in the patient selection domain according to the QUADAS-2 instrument, this source of bias exerts only a negligible influence on the validation phase of ML models.

ML employs supervised learning: within the independent validation set, predictions are first generated by the model and subsequently compared against the reference standard. The entire process is free from human intervention, preventing information leakage or overfitting. Consequently, the elevated selection bias inherent in case–control designs does not materially affect the pooled estimates of diagnostic performance reported in this quantitative synthesis.

Furthermore, the present investigation carries certain limitations in clinical applicability. Six studies were excluded from the meta-analysis because they reported only the AUC without furnishing adequate sample sizes, sensitivity, or specificity, preventing the construction of 2 × 2 contingency tables. These data were incomplete and did not meet the eligibility requirements of this synthesis. Early ML-related investigations commonly have insufficient disclosure of case numbers and incomplete reporting of key diagnostic indices. This circumstance, to some extent, compromises the completeness and comprehensiveness of the present findings and represents a shared methodological challenge for research in this domain.

Heterogeneity

Elucidating the sources of heterogeneity remains a critical challenge in current ML-associated systematic reviews. This meta-analysis likewise reveals substantial heterogeneity, underscoring the necessity of interpreting the findings with caution. Although subgroup analyses were undertaken, the capacity to account for potential sources of heterogeneity remains limited. Multiple factors across the included studies contribute to this situation. First, the studies originate from different regions, with variation in population characteristics, healthcare settings, diagnostic expertise, and the criteria applied for diagnosing CA, all of which may influence the pooled estimates. Second, discrepancies in imaging modality cannot be disregarded. Although ML models based on echocardiography were analyzed separately, heterogeneity in ultrasound acquisition and imaging protocols across device manufacturers and medical institutions may impact the observed diagnostic performance of ML models.

ML encompasses mature traditional inferential models such as logistic regression. Moreover, diagnostic performance varied substantially across different ML algorithms. The included studies employed random forests, support vector machines, neural networks, DL, and other techniques, with notable diversity in the architecture of models and configuration of parameters, further amplifying between-study heterogeneity. When more granular subgroup analyses by the subtype of CA were attempted, the relevant literature proved extremely sparse; most subgroups comprised fewer than four studies, failing to meet the operational prerequisites of the bivariate mixed-effects model and thus precluding further quantitative synthesis. This constitutes one of the major challenges currently confronting research on ML-based diagnosis of CA.

### Strengths and limitations of the study

This study is the first comprehensive review to assess the diagnostic performance of ML for CA, providing a basis for advancing the development of intelligent diagnostic tools. However, the study has the following limitations. First, the original studies included in this systematic review primarily focused on internal validation. Due to the limited external validation, the results of the external validation could not be presented. This may limit the interpretation of the findings. Second, although the included studies employed various imaging-based models, only a few utilized models constructed from images other than ECGs. Since bivariate mixed-effects models require at least four 2 × 2 diagnostic tables, we could not summarize the modeling effects of other images. Future research should explore the advantages of various imaging modeling approaches. Third, applying ML to diagnose CA may be subject to publication bias, which could limit the interpretation of the findings. Finally, substantial heterogeneity was found. The primary sources likely arise from variations in the clinical and imaging characteristics of the data used for modeling, as well as the diversity of the models themselves. Although subgroup analyses were conducted, the heterogeneity remained largely unexplained. This is attributable to underlying differences across models, including variations in the processing of images, acquisition protocols, and settings of parameters—even among studies using nominally the same type of models. Such heterogeneity represents a major challenge in conducting meta-analyses of ML studies. Future efforts will require larger sample sizes and a more extensive literature base to better elucidate and mitigate the sources of heterogeneity, which remains a key challenge for subsequent meta-analyses in this field.

### Outlook

The current investigation substantiates that ML exhibits relatively promising performance for diagnosing CA. Future research could develop high-performance ML models that integrate baseline demographic data and biomarkers of peripheral blood collected at hospital admission with imaging findings. Such integrated modeling could yield convenient, efficient, and rapid tools for early screening and risk prediction, thereby facilitating early identification and precise clinical intervention for CA.

## Conclusions

This study demonstrates that ML exhibits relatively satisfactory performance for noninvasively diagnosing CA, making it a valuable diagnostic aid in clinical practice. However, its detection performance needs to be further improved. The current findings provide evidence-based data for developing wearable devices and smart detection tools to diagnose CA in the future.

## Data Availability

The original contributions presented in the study are included in the article/Supplementary Material, further inquiries can be directed to the corresponding authors.
