# Supplementary material for: Machine learning-based methods in diagnosing cardiac amyloidosis: a meta-analysis
Source: Front Cardiovasc Med. 2026 Jul 3;13:1835652. doi: 10.3389/fcvm.2026.1835652 (PMC13375721; doi:10.3389/fcvm.2026.1835652)
Supplement: Supplementary file 1 [file Table1.docx]

**Appendix 1** Table S1 Literature search strategy

**1. PubMed**

| Search number | Query | Results |
| --- | --- | --- |
| #1 | ((((((((heart amyloidosis[Title/Abstract]) OR (amyloid cardiac disease[Title/Abstract])) OR (amyloid cardiac infiltration[Title/Abstract])) OR (amyloid cardiac involvement[Title/Abstract])) OR (amyloid heart disease[Title/Abstract])) OR (amyloidosis of the heart[Title/Abstract])) OR (cardiac amyloid involvement[Title/Abstract])) OR (cardiac amyloidosis[Title/Abstract])) OR (heart amyloidosis[Title/Abstract]) | 4428 |
| #2 | "Machine Learning"[Mesh] | 105,283 |
| #3 | (((((((((((((((((((((((((((((Transfer Learning[Title/Abstract]) OR (Deep learning[Title/Abstract])) OR (Ensemble Learning[Title/Abstract])) OR (artificial intelligence[Title/Abstract])) OR (random forest[Title/Abstract])) OR (neural network[Title/Abstract])) OR (neural networks[Title/Abstract])) OR (K-Nearest Neighbor[Title/Abstract])) OR (CNN[Title/Abstract])) OR (AlexNet[Title/Abstract])) OR (VGGNet[Title/Abstract])) OR (ResNet[Title/Abstract])) OR (GoogLeNet[Title/Abstract])) OR (Support vector machine[Title/Abstract])) OR (SVM[Title/Abstract])) OR (Gradient Boosting Machine[Title/Abstract])) OR (Nomogram[Title/Abstract])) OR (XGBoost[Title/Abstract])) OR (Adaboost[Title/Abstract])) OR (Decision tree[Title/Abstract])) OR (Naive Bayesian[Title/Abstract])) OR (Multilayer perceptron[Title/Abstract])) OR (Bayesian network[Title/Abstract])) OR (Prediction model[Title/Abstract])) OR (Risk model[Title/Abstract])) OR (Radiomics[Title/Abstract])) OR (Radiomic[Title/Abstract])) OR (radiogenomic[Title/Abstract])) OR (radiomics-based[Title/Abstract])) OR (Texture[Title/Abstract]) | 443,943 |
| #4 | ("Machine Learning"[Mesh]) OR ((((((((((((((((((((((((((((((Transfer Learning[Title/Abstract]) OR (Deep learning[Title/Abstract])) OR (Ensemble Learning[Title/Abstract])) OR (artificial intelligence[Title/Abstract])) OR (random forest[Title/Abstract])) OR (neural network[Title/Abstract])) OR (neural networks[Title/Abstract])) OR (K-Nearest Neighbor[Title/Abstract])) OR (CNN[Title/Abstract])) OR (AlexNet[Title/Abstract])) OR (VGGNet[Title/Abstract])) OR (ResNet[Title/Abstract])) OR (GoogLeNet[Title/Abstract])) OR (Support vector machine[Title/Abstract])) OR (SVM[Title/Abstract])) OR (Gradient Boosting Machine[Title/Abstract])) OR (Nomogram[Title/Abstract])) OR (XGBoost[Title/Abstract])) OR (Adaboost[Title/Abstract])) OR (Decision tree[Title/Abstract])) OR (Naive Bayesian[Title/Abstract])) OR (Multilayer perceptron[Title/Abstract])) OR (Bayesian network[Title/Abstract])) OR (Prediction model[Title/Abstract])) OR (Risk model[Title/Abstract])) OR (Radiomics[Title/Abstract])) OR (Radiomic[Title/Abstract])) OR (radiogenomic[Title/Abstract])) OR (radiomics-based[Title/Abstract])) OR (Texture[Title/Abstract])) | 473,865 |
| #5 | (((((((((heart amyloidosis[Title/Abstract]) OR (amyloid cardiac disease[Title/Abstract])) OR (amyloid cardiac infiltration[Title/Abstract])) OR (amyloid cardiac involvement[Title/Abstract])) OR (amyloid heart disease[Title/Abstract])) OR (amyloidosis of the heart[Title/Abstract])) OR (cardiac amyloid involvement[Title/Abstract])) OR (cardiac amyloidosis[Title/Abstract])) OR (heart amyloidosis[Title/Abstract])) AND (("Machine Learning"[Mesh]) OR ((((((((((((((((((((((((((((((Transfer Learning[Title/Abstract]) OR (Deep learning[Title/Abstract])) OR (Ensemble Learning[Title/Abstract])) OR (artificial intelligence[Title/Abstract])) OR (random forest[Title/Abstract])) OR (neural network[Title/Abstract])) OR (neural networks[Title/Abstract])) OR (K-Nearest Neighbor[Title/Abstract])) OR (CNN[Title/Abstract])) OR (AlexNet[Title/Abstract])) OR (VGGNet[Title/Abstract])) OR (ResNet[Title/Abstract])) OR (GoogLeNet[Title/Abstract])) OR (Support vector machine[Title/Abstract])) OR (SVM[Title/Abstract])) OR (Gradient Boosting Machine[Title/Abstract])) OR (Nomogram[Title/Abstract])) OR (XGBoost[Title/Abstract])) OR (Adaboost[Title/Abstract])) OR (Decision tree[Title/Abstract])) OR (Naive Bayesian[Title/Abstract])) OR (Multilayer perceptron[Title/Abstract])) OR (Bayesian network[Title/Abstract])) OR (Prediction model[Title/Abstract])) OR (Risk model[Title/Abstract])) OR (Radiomics[Title/Abstract])) OR (Radiomic[Title/Abstract])) OR (radiogenomic[Title/Abstract])) OR (radiomics-based[Title/Abstract])) OR (Texture[Title/Abstract]))) | 143 |

**2. Cochrane**

| Search number | Query | Results |
| --- | --- | --- |
| #1 | (amyloid cardiac disease):ti,ab,kw OR (amyloid cardiac infiltration):ti,ab,kw OR (amyloid cardiac involvement):ti,ab,kw OR (amyloid heart disease):ti,ab,kw OR (amyloidosis of the heart):ti,ab,kw |  |
| #2 | (cardiac amyloid involvement):ti,ab,kw OR (cardiac amyloidosis):ti,ab,kw OR (heart amyloidosis):ti,ab,kw | 391 |
| #3 | #1 or #2 | 575 |
| #4 | MeSH descriptor: [Machine Learning] explode all trees | 1222 |
| #5 | (Transfer Learning):ti,ab,kw OR (Deep learning):ti,ab,kw OR (Ensemble Learning):ti,ab,kw OR (artificial intelligence):ti,ab,kw OR (random forest):ti,ab,kw | 7415 |
| #6 | (neural network):ti,ab,kw OR (neural networks):ti,ab,kw OR (K-Nearest Neighbor):ti,ab,kw OR (CNN):ti,ab,kw OR (AlexNet):ti,ab,kw | 4210 |
| #7 | (VGGNet):ti,ab,kw OR (ResNet):ti,ab,kw OR (GoogLeNet):ti,ab,kw OR (Support vector machine):ti,ab,kw OR (SVM):ti,ab,kw | 727 |
| #8 | (Gradient Boosting Machine):ti,ab,kw OR (Nomogram):ti,ab,kw OR (XGBoost):ti,ab,kw OR (Adaboost):ti,ab,kw OR (Decision tree):ti,ab,kw | 3240 |
| #9 | (Naive Bayesian):ti,ab,kw OR (Multilayer perceptron):ti,ab,kw OR (Bayesian network):ti,ab,kw OR (Prediction model):ti,ab,kw OR (Risk model):ti,ab,kw | 38289 |
| #10 | (Radiomics):ti,ab,kw OR (Radiomic):ti,ab,kw OR (radiogenomic):ti,ab,kw OR (radiomics-based):ti,ab,kw OR (Texture):ti,ab,kw | 3194 |
| #11 | #4 or #5 or #6 or #7 or #8 or #9 or #10 | 52066 |
| #12 | #3 and #11 | 38 |

**3. Embase**

| Search number | Query | Results |
| --- | --- | --- |
| #1 | 'heart amyloidosis'/exp | 10016 |
| #2 | 'amyloid cardiac disease':ab,ti OR 'amyloid cardiac infiltration':ab,ti OR 'amyloid cardiac involvement':ab,ti OR 'amyloid heart disease':ab,ti OR 'amyloidosis of the heart':ab,ti OR 'cardiac amyloid involvement':ab,ti OR 'cardiac amyloidosis':ab,ti OR 'heart amyloidosis':ab,ti | 8074 |
| #3 | #1 OR #2 | 11132 |
| #4 | 'machine learning'/exp | 662636 |
| #5 | 'transfer learning':ab,ti OR 'deep learning':ab,ti OR 'ensemble learning':ab,ti OR 'artificial intelligence':ab,ti OR 'random forest':ab,ti OR 'neural network':ab,ti OR 'neural networks':ab,ti OR 'k-nearest neighbor':ab,ti OR cnn:ab,ti OR alexnet:ab,ti OR vggnet:ab,ti OR resnet:ab,ti OR googlenet:ab,ti OR 'support vector machine':ab,ti OR svm:ab,ti OR 'gradient boosting machine':ab,ti OR nomogram:ab,ti OR xgboost:ab,ti OR adaboost:ab,ti OR 'decision tree':ab,ti OR 'naive bayesian':ab,ti OR 'multilayer perceptron':ab,ti OR 'bayesian network':ab,ti OR 'prediction model':ab,ti OR 'risk model':ab,ti OR radiomics:ab,ti OR radiomic:ab,ti OR radiogenomic:ab,ti OR 'radiomics based':ab,ti OR texture:ab,ti | 529587 |
| #6 | #4 OR #5 | 911078 |
| #7 | #3 AND #6 | 436 |

**4. Web of science**

| Search number | Query | Results |
| --- | --- | --- |
| #1 | TS=(heart amyloidosis OR amyloid cardiac disease OR amyloid cardiac infiltration OR amyloid cardiac involvement OR amyloid heart disease OR amyloidosis of the heart OR cardiac amyloid involvement OR cardiac amyloidosis OR heart amyloidosis) | 12000 |
| #2 | TS=(machine learning OR Transfer Learning OR Deep learning OR Ensemble Learning OR artificial intelligence OR random forest OR neural network OR neural networks OR K-Nearest Neighbor OR CNN OR AlexNet OR VGGNet OR ResNet OR GoogLeNet OR Support vector machine OR SVM OR Gradient Boosting Machine OR Nomogram OR XGBoost OR Adaboost OR Decision tree OR Naive Bayesian OR Multilayer perceptron OR Bayesian network OR Prediction model OR Risk model OR Radiomics OR Radiomic OR radiogenomic OR radiomics-based OR Texture) | 4280547 |
| #3 | #2 AND #1 | 710 |
