# Supplementary material for: Machine learning-based methods in diagnosing cardiac amyloidosis: a meta-analysis
Source: Front Cardiovasc Med. 2026 Jul 3;13:1835652. doi: 10.3389/fcvm.2026.1835652 (PMC13375721; doi:10.3389/fcvm.2026.1835652)
Supplement: Supplementary file 2 [file Table2.docx]

**Appendix 2** The diagnostic four-fold table of machine learning for detecting CA in studies

| Study | Subject | Data_set | Sensitivity | Specificity | tp | fp | fn | tn |
| --- | --- | --- | --- | --- | --- | --- | --- | --- |
| Zhou XY(2022) | CA | Training set | 0.8600 | 0.8400 | 44 | 7 | 7 | 39 |
| Yue X(2022) | AL-CA | Training set | 0.8130 | 1.0000 | 20 | 0 | 4 | 17 |
| Lo Iacono F(2023) | CA | Training set | 0.9300 | 0.9300 | 14 | 1 | 1 | 14 |
| Huang S(2022) | CA | Training set | 0.7570 | 0.9070 | 76 | 20 | 24 | 197 |
| Delbarre MA(2023) | CA | Training set | 0.9890 | 0.9940 | 278 | 17 | 3 | 2750 |
| Barbieri A(2023) | CA | Training set | 0.8685 | 1.0000 | 9 | 0 | 1 | 177 |
| Zhou XY(2022) | CA | Validation set | 0.7900 | 0.8600 | 22 | 2 | 6 | 12 |
| Zhou XY(2022) | CA | Validation set | 0.9000 | 0.8000 | 37 | 4 | 4 | 16 |
| Zhang X(2023) | CA | Validation set | 0.7850 | 0.6880 | 12 | 22 | 3 | 50 |
| Zhang J(2018) | AL-CA | Validation set | 0.8419 | 0.7317 | 59 | 13 | 11 | 37 |
| Schrutka L(2021) | CA | Validation set | 0.8900 | 0.8000 | 27 | 10 | 3 | 40 |
| Huang S(2022) | CA | Validation set | 0.7120 | 0.8330 | 31 | 16 | 12 | 77 |
| Haimovich JS(2023) | CA | Validation set | 0.9277 | 0.8774 | 146 | 1998 | 11 | 14296 |
| Goto S(2021) | CA | Validation set | 0.8360 | 0.8679 | 92 | 44 | 18 | 287 |
| Goto S(2021) | CA | Validation set | 0.8061 | 0.8569 | 37 | 46 | 9 | 277 |
| Goto S(2021) | CA | Validation set | 0.8809 | 0.9228 | 104 | 9 | 14 | 112 |
| Goto S(2021) | AATR-CA | Validation set | 0.9120 | 0.8805 | 19 | 11 | 2 | 78 |
| Goto S(2021) | AATR-CA | Validation set | 0.9739 | 0.9827 | 9 | 1 | 0 | 36 |
| Goto S(2021) | AATR-CA | Validation set | 0.8816 | 0.9277 | 40 | 5 | 5 | 68 |
| Goto S(2021) | AL-CA | Validation set | 0.8686 | 0.8503 | 15 | 14 | 2 | 79 |
| Goto S(2021) | AL-CA | Validation set | 0.8373 | 0.7322 | 9 | 9 | 2 | 26 |
| Goto S(2021) | AL-CA | Validation set | 0.9129 | 0.8719 | 6 | 14 | 1 | 97 |
| Germain P(2022) | CA | Validation set | 0.7700 | 0.7100 | 37 | 14 | 11 | 35 |
| Germain P(2022) | CA | Validation set | 0.8570 | 0.7760 | 232 | 60 | 39 | 206 |
| Delbarre MA(2023) | CA | Validation set | 0.9610 | 0.9950 | 98 | 8 | 4 | 1523 |
| Cuddy SA(2022) | ATTR-CA | Validation set | 0.7500 | 0.7750 | 173 | 44 | 57 | 151 |
| Arvanitis M(2017) | ATTR-CA | Validation set | 0.9680 | 0.5475 | 9 | 12 | 0 | 15 |
| Arvanitis M(2017) | CA | Validation set | 1.0000 | 0.5632 | 9 | 12 | 0 | 15 |
| Arana-Achaga X(2023) | ATTR-CA | Validation set | 0.8916 | 0.8217 | 96 | 21 | 12 | 98 |
| Arana-Achaga X(2023) | CA | Validation set | 0.7747 | 0.7491 | 330 | 118 | 96 | 351 |
| Antonopoulos AS(2021) | CA | Validation set | 0.9148 | 0.3967 | 26 | 73 | 2 | 48 |
| Martini N(2020) | CA | Validation set | 0.8600 | 0.8300 | 33 | 1 | 5 | 5 |
| Asan Agibetov(2021) | CA | Validation set | 0.9700 | 0.8100 | 80 | 80 | 2 | 340 |
| Tsang C(2023) | ATTR-CA | Validation set | 0.6700 | 0.8900 | 9969 | 1585 | 4993 | 13377 |
| Slivnick JA(2025) | CA | Validation set | 0.8500 | 0.9300 | 507 | 149 | 90 | 1973 |
| Tohyama T(2025) | CA | Validation set | 1.0000 | 1.0000 | 25 | 0 | 0 | 30 |
| Chang RS(2024) | CA | Validation set | 0.8200 | 0.7300 | 522 | 801 | 114 | 2166 |
| Hong Z(2025) | CA | Validation set | 0.8300 | 0.9400 | 22 | 1 | 5 | 22 |
| Hourmozdi J(2025) | ATTR-CA | Validation set | 0.8200 | 0.8800 | 144 | 362 | 32 | 2654 |
| Bargagna F(2025) | CA | Validation set | 0.9900 | 0.9900 | 45 | 1 | 0 | 56 |
| Bargagna F(2025) | ATTR-CA | Validation set | 0.7600 | 0.8000 | 24 | 14 | 8 | 56 |
| Vrudhula A(2024) | CA | Validation set | 0.6100 | 0.7800 | 4572 | 290574 | 2935 | 1036249 |
| Pan Y(2024) | CA | Validation set | 0.9200 | 0.9500 | 240 | 315 | 21 | 5987 |
